# Supplementary material for: Temporal predictions shape somatosensory perception
Source: Nat Commun. 2026 Apr 14;17:3476. doi: 10.1038/s41467-026-71600-y (PMC13079782; doi:10.1038/s41467-026-71600-y)
Supplement: Supplementary file 1 — Supplementary Information [file 41467_2026_71600_MOESM1_ESM.pdf]

Supplementary Information

Title

Temporal Predictions Shape Somatosensory Perception

Author List

Andreas Strube[1,‡,*] and Christian Büchel[1,*]

Affiliations

[1]Department of Systems Neuroscience, University Medical Center Hamburg-Eppendorf, 20246 Hamburg, Germany

[‡]Present address: Center for Depression, Anxiety and Stress Research, Department of Psychiatry, McLean Hospital, Harvard Medical School, Boston, MA, USA

*Corresponding Authors:
Andreas Strube: andreasstrube@outlook.com
Christian Büchel: buechel@uke.de

Full LME Model Specification

Linear mixed-effects model fit by ML

Model information:
Number of observations 10080
Fixed effects coefficients 8
Random effects coefficients 175
Covariance parameters 16

Formula:
Rating ~ 1 + StimMod*StimLat + StimMod*Expectation + StimMod*PE + (1 + StimMod + StimLat + Expectation + PE | Subject)

Model fit statistics:

AIC	BIC	LogLikelihood	Deviance
8951.7	9125	-4451.9	8903.7

Fixed effects coefficients (95% CIs):

Name	Estimate	SE	tStat	DF	pValue	Lower	Upper
{'(Intercept)' }	-0.84662	0.063491	-13.335	10072	3.1893e-40	-0.97108	-0.72217
{'StimMod_1' }	1.6932	0.10032	16.878	10072	4.8133e-63	1.4966	1.8899
{'StimLat' }	0.00088724	0.0061789	0.14359	10072	0.88583	-0.011225	0.012999
{'Expectation' }	0.020329	0.0063191	3.2171	10072	0.001299	0.0079426	0.032716
{'PE' }	-0.001038	0.005239	-0.19812	10072	0.84296	-0.011307	0.0092316
{'StimMod_1:StimLat' }	-0.002039	0.0085024	-0.23981	10072	0.81048	-0.018705	0.014627
{'StimMod_1:Expectation'}	-0.0023696	0.0085024	-0.2787	10072	0.78048	-0.019036	0.014297
{'StimMod_1:PE' }	0.0032102	0.0073633	0.43597	10072	0.66287	-0.011223	0.017644

Random effects covariance parameters (95% CIs):
Group: Subject (35 Levels)

Name1	Name2	Type	Estimate	Lower	Upper
{'(Intercept)'}	{'(Intercept)'}	{'std' }	0.37435	0.30739	0.4559
{'StimMod_1' }	{'(Intercept)'}	{'corr'}	-0.76458	NaN	NaN
{'StimLat' }	{'(Intercept)'}	{'corr'}	-0.5422	NaN	NaN
{'Expectation'}	{'(Intercept)'}	{'corr'}	0.59156	NaN	NaN
{'PE' }	{'(Intercept)'}	{'corr'}	0.28833	0.26635	0.31001
{'StimMod_1' }	{'StimMod_1' }	{'std' }	0.59193	0.48603	0.72091
{'StimLat' }	{'StimMod_1' }	{'corr'}	0.72751	NaN	NaN
{'Expectation'}	{'StimMod_1' }	{'corr'}	-0.45948	NaN	NaN
{'PE' }	{'StimMod_1' }	{'corr'}	-0.19007	-0.25402	-0.12446
{'StimLat' }	{'StimLat' }	{'std' }	0.0084372	0.0029592	0.024056
{'Expectation'}	{'StimLat' }	{'corr'}	0.22668	0.22265	0.23071
{'PE' }	{'StimLat' }	{'corr'}	0.52239	0.4618	0.57812
{'Expectation'}	{'Expectation'}	{'std' }	0.011511	0.0049691	0.026665
{'PE' }	{'Expectation'}	{'corr'}	0.94105	0.93421	0.94719
{'PE' }	{'PE' }	{'std' }	0.003441	0.00027652	0.042819

Group: Error

Name	Estimate	Lower	Upper
{'Res Std'}	0.36962	0.36452	0.37478

Supplementary Bayesian Integration Model Analysis

Methods

For exploratory model-based analysis of our intensity VAS rating data, we designed Bayesian integration models of somatosensation (Figure Supplementary Figure 1; see Büchel et al. 2014 for a review) in accordance with the expectation intensity shift model (Supplementary Figure 1b) and the expectation precision modulation model (Supplementary Figure 1c). In the Bayesian formulation of pain perception, Bayes' theorem is used to estimate the level of perceived stimuli, taking precision-weighted prior experiences into account (Supplementary Figure 1a; Equation 1).

Our goal was to describe and formalize a perceptual process in which subjective ratings reflect a precision-weighted integration of prior expectations and sensory input. Accordingly, we did not apply Bayes' theorem in the inferential form but used a Bayesian observer model to compute an internal perceptual estimate, assuming Gaussian prior and likelihood distributions. This approach is common in perceptual modeling (Büchel et al., 2014; Ernst & Banks, 2002; Körding & Wolpert, 2004; Strube et al., 2023). Our model does not infer parameters from data but models the subjective estimate of pain intensity as the posterior mean resulting from the integration of prior expectation and sensory input.

Formally, the Bayesian integration model integrates a prior - representing internal top-down processes, such as expectations - with a likelihood - representing bottom-up sensory input - to estimate a posterior - the percept. Both the prior and the likelihood were approximated by normal distributions allowing for an analytical integration using normal-normal conjugate priors to estimate the normal posterior:

Eq1)

$$\mu_{posterior} = \frac{\mu_{prior} * \sigma^2_{likelihood} + \mu_{likelihood} * \sigma^2_{prior}}{\sigma^2_{likelihood} + \sigma^2_{prior}}$$

$$\sigma^2_{posterior} = \frac{\sigma^2_{likelihood} * \sigma^2_{prior}}{\sigma^2_{likelihood} + \sigma^2_{prior}}$$

With respect to the behavioral intensity rating data, our model predicted the distribution of intensity ratings (posterior) by integrating an empirically-informed prior resulting from the expected latency from cue offset to stimulus onset of $t = 0$ as a prior (mean and variance derived from cold and heat intensity VAS ratings for cold and heat conditions, respectively) with a likelihood. The individual estimate of the likelihood was also derived from intensity ratings with an expected latency from cue offset to stimulus onset of $t = 0$. Gaussian approximation of the rating data was performed by fitting a Gaussian cumulative probability density function to the cumulative sum of the ratings using a robust grid search.

With respect to the behavioral intensity rating data, our model predicted the distribution of perceived intensity ratings (posterior) by integrating the prior that reflects the expected latency from cue offset to stimulus onset of $t = 0$. This prior was not directly observed but was

constructed based on intensity VAS ratings for cold and heat conditions under this baseline latency, serving as a theoretical benchmark for expectation.

Similarly, the likelihood was also modeled from VAS ratings under t = 0 latency and reflects the assumed sensory input distribution. These distributions served as input to the model, whereas the posterior represents the modeled perceptual outcome (i.e., predicted intensity ratings) for the different experimental conditions. Gaussian approximation of the rating data was performed by fitting a cumulative Gaussian function to the cumulative sum of the observed ratings using a robust grid search procedure.

For a expectation precision modulation to have the influence to shift the posterior towards the likelihood, we needed to shift the prior to lower values by a negative offset (which was accordingly positively applied to the likelihood) which was individually fitted by a free parameter in our model - for example, when the mean rating of trials with instant stimulation expectation (0s delay between cue and stimulus) was at 70, the prior mean could take values lower than 70 while the likelihood could accordingly take values higher than 70, while keeping the prior-posterior and likelihood-posterior mean distances equal. We included the quadratic parameter p_offset (see Eq. 2, 3 and 4). Additionally, we multiplied the prior and likelihood variance by 2 and thus ensured a correct posterior for instant stimulation trials by Bayesian integration.

This modeling approach allows us to formally compare how changes in latency expectations affect perception by shifting the prior or its precision, without claiming that the priors or likelihoods were directly measured. Rather, they serve as theoretically informed constructs derived from a stable reference condition (t = 0), enabling mechanistic (i.e.: can our results be explained by a shift or a precision modulation?) inference about expectation-related modulation in pain perception. Thus, the prior and likelihood values in our models were not directly measured but constructed as theoretically informed possible approximations. Our goal was not to recover true internal parameters, but to test whether a shift in expectations or a change in expectation precision could mechanistically explain the observed effects within a Bayesian integration model.

For the estimation of the posterior parameters in early and late stimulation trials we created two models, based on a shift of the prior and a modulation of the prior precision. For the expectation intensity shift model, we included a free parameter p_shift to enable a prior shift which was increasing linearly with higher cue-stimulus delays (Supplementary Figure 1b; Eq2):

Eq2)

$$\mu_{posterior} = \frac{(\mu_{prior} - p_{offset}^{2} + p_{shift}) * \sigma^2_{likelihood} + (\mu_{likelihood} + p_{offset}^{2}) * \sigma^2_{prior}}{\sigma^2_{likelihood} + \sigma^2_{prior}}$$

$$\sigma^2_{posterior} = \frac{\sigma^2_{likelihood} * \sigma^2_{prior}}{\sigma^2_{likelihood} + \sigma^2_{prior}}$$

For the expectation precision modulation model we included a free parameter p_precision to represent a modulation of variance by longer cue-stimulus delays. Under this model, posterior parameters are estimated by the following equations (Equation 3; see also Supplementary Figure 1c):

Eq3)

$$\mu_{posterior} = \frac{(\mu_{prior} - p_{offset}{}^{2}) * \sigma^2_{likelihood} + (\mu_{likelihood} + p_{offset}{}^{2}) * (\sigma^2_{prior} * p_{precision})}{\sigma^2_{likelihood} + (\sigma^2_{prior} * p_{precision})}$$

$$\sigma^2_{posterior} = \frac{\sigma^2_{likelihood} * (\sigma^2_{prior} * p_{precision})}{\sigma^2_{likelihood} + (\sigma^2_{prior} * p_{precision})}$$

We used variational Bayesian inference to estimate the parameters of all models using the VBA toolbox (Daunizeau et al., 2014) for Matlab (R2025a). The estimated parameters included for the expectation intensity shift model, p_offset and p_shift, and, for the precision-modulation model, p_offset and p_precision. Parameter estimation in VBA is based on the minimization of variational free energy, which serves as the optimization criterion. This approach approximates the true posterior density of the parameters by optimizing a variational bound on model evidence.

Note that we also tested a null model (Equation 1) in which the shift parameter was "constrained" through its priors pshift ~ Normal(0, 1e-20), and models in which we had separate p_shift and p_precision parameters estimates for cold and heat stimulation trials, and an additional model where we captured the idea that the precision could increase with higher latency (p_offset signs were reversed, shifting the prior mean to higher and the likelihood mean to lower values, respectively). Given our behavioral intensity VAS rating data (i.e. empirical posterior), VBA recovers an approximation to both the posterior density on unknown variables (p_precision and p_shift for the precision models and the shift model, respectively, and p_offset for both models) and the log model evidence (which is used for model comparison). We used a random effects (RFX) (Rigoux et al., 2014; Stephan et al., 2009) Bayesian model selection approach to estimate the overall posterior model probability across subjects. We estimated the protected exceedance probability as a metric for the Bayesian model comparison of all candidate models.

We also considered a scenario where increasing the precision of the prior would shift the posterior mean towards higher values. We conducted additional analyses using a fixed p_offset parameter set to realize an offset of the prior and likelihood by 5, 10, and 20 VAS points. Finally, we tested a model with an ideal offset, based on the parameter estimate for each individual subject which was fixed in model comparison.

Bayesian Integration - Modeling Details

We formalized pain perception in terms of Bayesian inference, where an internal prior expectation about stimulus intensity is combined with sensory evidence (likelihood) to generate a posterior percept.

Modelling of prior expectations and likelihood with the given data:

The prior derived from each participant's ratings in the instant expectation condition, shifted depending on the model. As σ^2, in the case of the likelihood and the prior, we used σ^2 of the instant expectation condition as the base condition. To assure, in Bayesian integration, that the resulting posterior equals the instant likelihood condition, we multiplied σ^2 in both cases with 2.

Note that we estimated the p_offset as an estimated parameter and included it in Eq3. (see Eq. 3; here, $\mu_{\text{prior}} = \mu_{\text{instantExpectation}}$ and $\mu_{\text{likelhihood}} = \mu_{\text{instantExpectation}}$). Thus, both μ_prior and μ_likelihood were identical, and were offset with a different sign by p_offset, i.e., which means for the prior,

$$\mu_{\text{prior}} = \mu_{\text{instantExpectation}} - (p_{\text{offset}})^2$$

$$\sigma^2_{\text{prior}} = 2 * \sigma^2_{\text{instantEpectation}}$$

and for the likelihood, which is defined by a complementary shift relative to the prior:

$$\mu_{\text{likelihood}} = \mu_{\text{instantExpectation}} + (p_{\text{offset}})^2$$

$$\sigma^2_{\text{likelihood}} = 2 * \sigma^2_{\text{instantEpectation}}$$

Parameters Estimated

Depending on the model, we estimated different sets of free parameters with the following Gaussian prior distributions:

Expectation intensity shift model:
p_shift: linear increase in prior shift with longer expected delays (Prior: mean = 0 / var = 1000)
p_offset : quadratic offset applied symmetrically to prior and likelihood / (Prior mean = 0 / var = 1000)

Expectation precision modulation model:
p_precision: multiplicative factor on σ^2 / (Prior mean = 1 / var = 1000)
p_offset : quadratic offset as above / (Prior mean = 0 / var = 1000)

Null model: All parameters are constrained to zero or one (prior distribution fixed at instant condition).

p_shift: linear increase in prior shift with longer expected delays / mean = 0 / var = eps
p_offset : quadratic offset applied symmetrically to prior and likelihood / mean = 0 / var = eps
p_precision: multiplicative factor on σ^2 / mean = 1 / var = eps

We also tested two-parameter variants with separate p_shift or p_precision for painful heat and non-painful cold with the same prior distributions. We also considered a expectation precision modulation model where the prior expectations could sharpen, here, p_offset could only take positive values (shifting the prior to higher values than the likelihood). In all cases, the

protected exceedance probability was at $\varphi \approx 1$ for the expectation intensity shift model compared with $\varphi \approx 0$ for all other models.

Finally, as the expectation intensity shift model worked regardless of a p_offset, whereas the expectation precision modulation model needs to shift the prior and likelihood in order to "create" a shift of the posterior by precision modulation, we wanted to control if our results were dependent on the free parameter p_offset and fixed it to 3 different values (5, 10, 20) and in each case, the expectation intensity shift model won. Finally, we also tested an ideal offset (based on the parameter estimate for each subject of p_offset for the expectation precision modulation model, and again, the expectation intensity shift model won, i.e., in each case, the protected exceedance probability was at $\varphi \approx 1$ for the expectation intensity shift model compared with $\varphi \approx 0$ for the expectation precision modulation model.

Results: Expectation Precision vs Intensity Expectations

We explored two potential mechanisms within a Bayesian integration model of somatosensory perception: whether the observed effects arise from a shift in prior expectations or from changes in the precision of these expectations (Büchel et al., 2014; Camerone et al., 2025; Grahl et al., 2018; Habermann et al., 2024; Strube et al., 2023; Strube & Büchel, 2023).

In this explorative Bayesian integration model, both interoceptive sensory information as well as prior expectations are weighted in terms of their respective precision and intensity (integration). This has been demonstrated to play a mechanistic role in control and agency (Habermann et al., 2024; Habermann & Büchel, 2025; Strube et al., 2023) as well as in conditioned pain expectation (Grahl et al. 2018). In this model, the somatosensory percept resembles this integration of expectations and sensory information. The modulation of somatosensation based on temporal expectations might therefore also be explainable by a change in expectation or sensory information. This model not only considers the mean of sensory information and expectation, but also their precisions. Such models build upon a theoretical Bayesian framework of perception, not Bayesian inference in the classical sense: they specify how expectations might mechanistically shape pain experience via either an increase in prior intensity (e.g., due to aversive effects of dread) or a decrease in prior precision (e.g., due to temporal uncertainty). Bayesian integration models of perception are widely used in cognitive neuroscience to formalize how the brain integrates prior beliefs with sensory evidence (e.g. Büchel et al., 2014; Ernst & Banks, 2002; Grahl et al., 2018; Körding & Wolpert, 2004; Strube et al., 2023).

We exploratory applied computational modelling to our behavioral data and compared Bayesian integration models of pain perception (see Supplementary Figure 1). We used the Bayesian integration model of pain perception as the basic model (Supplementary Figure 1a). In the expectation intensity shift model, we included an additional free parameter p_shift which allowed for a shift of the expectation (prior) (Supplementary Figure 1b). This is represented in a shift of the prior mean to higher values with a larger expected latency, representing cognitive and affective responses such as potentially dread, which lead to a higher intensity percept. In the expectation precision modulation model, we introduced a free parameter (p_precision) that modulates the precision of the prior expectation (Supplementary Figure 1c). Reducing prior precision effectively "loosens" the expectation, making it less constraining on the perceptual inference process. As a result, the posterior distribution is more strongly influenced by the sensory input. This leads to a posterior mean shifted toward greater pain intensity, assuming a higher sensory input than expectations. Importantly, unlike a simple shift of the prior mean, a

reduced precision also reduces the overall posterior precision, because the posterior inherits its precision from the combination of prior and sensory likelihood. Thus, precision modulation predicts both, higher pain ratings and less certainty in these ratings, offering a qualitatively distinct mechanism from the expectation intensity shift model.

Note that these values are not meant to reflect empirically observed parameters (e.g. expectations were not specifically measured), but rather serve as theoretically plausible constructs that allow us to isolate and compare the effects of a shift or precision modulation mechanism within a Bayesian integration framework.

An expectation intensity shift would therefore represent actual higher intensity expectations, as for example induced by dread, or other affective or cognitive responses that manifest as intensity expectations. In contrast, an expectation precision modulation (Supplementary Figure 1c) would represent induced uncertainty, for example by worse temporal estimates of the anticipated waiting period, following Weber's law (Fechner, 1860; Grabenhorst et al., 2019; Haigh et al., 2021). Here, one would assume, temporal estimations are getting worse with increased time periods. For example: We can provide a reliable estimate of one second with a confidence interval of milliseconds, whereas the estimation of six hours is associated with a confidence interval of minutes or even hours. Therefore, we hypothesized that a longer anticipated waiting period could be associated with higher contextual uncertainty, which can be translated to a reduced prior precision. Although the predictive cue in our experiment explicitly signals the expected timing of the stimulation and its information should not "decay" over the short latencies used, it is likely that temporal expectation is subject to internal uncertainty during the anticipation phase. In particular, we hypothesized that longer anticipated delays entail more contextual or cognitive uncertainty about when exactly the stimulation will occur (Grabenhorst et al., 2019).

We used a random effects (RFX) Bayesian model selection approach (Rigoux et al., 2014; Stephan et al., 2009; see Methods and Supplementary Information for modelling details) to estimate the overall posterior model probability across subjects (i.e., estimating if a perceptual Bayesian integration model with a prior shift or a precision modulation captures the data better). The RFX model protected exceedance probability was at $\varphi \approx 1$ for the expectation intensity shift model compared with $\varphi \approx 0$ for the expectation precision modulation model. Hence, we see clear evidence for the expectation intensity shift model over expectation precision modulation model (see Supplementary Figure 2 for parameter estimates across participants and parameter correlations with behavioral outcomes). The expectation intensity shift model also outperforms all other models, i.e., a null model without free parameters (i.e., setting the shift parameter to 0), and models separating parameters for cold and pain stimuli (see Methods for details; see Supplementary Figure 3 for results with simulated data). To address the theoretical possibility that longer expected delays might also increase expectation precision (rather than decrease it), we implemented and tested an additional model against all other models, in which prior precision increased with increasing expected latency. This could correspond to the idea that fewer alternative outcomes are possible with longer delays, leading to stronger predictions (for example, within a trial, when 2s have passed, stimulation after 4s or catch are the only options). However, this model did not outperform the intensity shift model in our comparison ($\varphi \approx 0$).

Thus, Bayesian model selection provides strong evidence in favor of the expectation intensity shift model over all other candidate models, suggesting that the increase in intensity ratings can

be explained by an increase of top-down prior intensity expectations, as compared to a modulation of prior expectation precision.

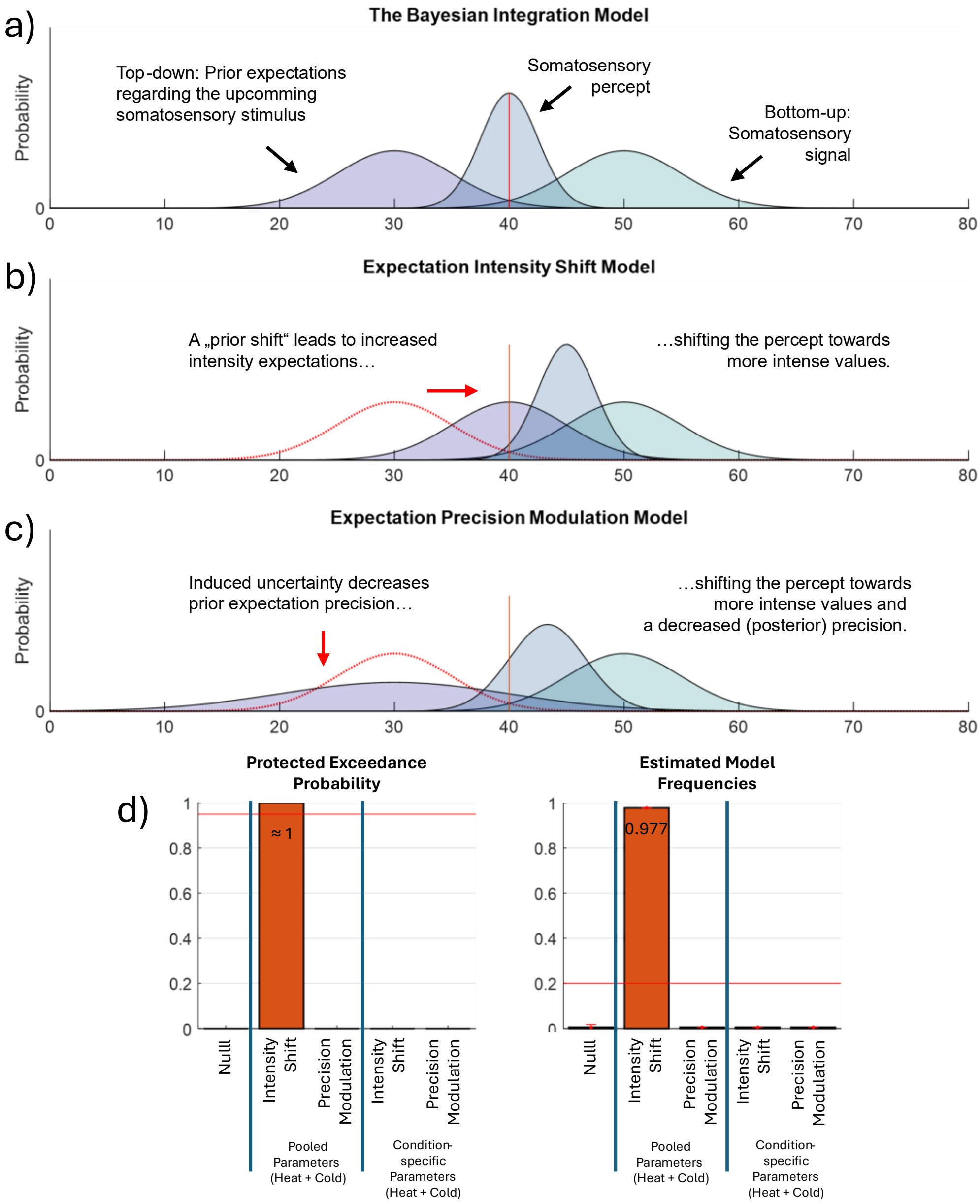

Supplementary Figure 1. The Bayesian integration model. Hypothetical gaussian distributions represent prior intensity expectations (purple) and somatosensory input (cyan), which are integrated in a precision-weighted manner to produce the somatosensory percept represented by a posterior distribution (blue). Expectations are generated and integrated with the sensory input, resulting in a shift of the somatosensory percept towards the expectations on a visual analog scale (VAS). b) The expectation intensity shift model: When a prior mean shift occurs (i.e. the expectation is shifted towards higher VAS values reflecting higher intensity expectations), it results in increased pain (indicated by a higher mean posterior value). c) The expectation precision modulation model: Alternatively, a decrease of prior precision may serve as a possible mechanism through which temporal expectations induce uncertainty and modulate somatosensory perception. In this model, lower prior precision reduces the influence of expectations and increases the relative weight of the sensory input. When the actual sensory

input is more painful than expected, the posterior distribution is shifted toward higher pain intensity (as in 4b). However, this mechanism additionally leads to a lower posterior precision, as the posterior “inherits” its precision from both prior and sensory likelihood. Note that in this figure, the prior mean is assumed to be lower than the likelihood mean. However, one could also imagine a scenario in which the prior mean is higher than the likelihood mean. In such a case, increasing the precision of the prior would shift the posterior mean towards higher values. d) Model frequencies and protected exceedance probabilities show that our data (n = 35) strongly favor the expectation intensity shift model over the expectation precision modulation model.

Correlation with behavioral ratings (expectation effect)

As a control analysis, we correlated our parameter estimates for p_shift and p_precision with the expectation effect, i.e.:

p_shift was strongly correlated with our expectation effect, $p < .001$:

Linear contrast Expectation ~ p_shift: $r=0.880$, $p<0.001$

p_precision showed a tendency to correlate with our expectation effect. $p < .1$:

Linear contrast Expectation ~ p_precision: $r=0.287$, $p=0.095$

See Supplementary Figure 2 for parameter estimates across participants. See Supplementary Table 1 for the posterior parameter distributions (Gaussian mean and standard deviation) of the main models, i.e., for p_shift, p_precision, and p_offset (for the expectation precision modulation model; in the expectation intensity shift model, p_offset was fixed at mean = 0, SD = 0).

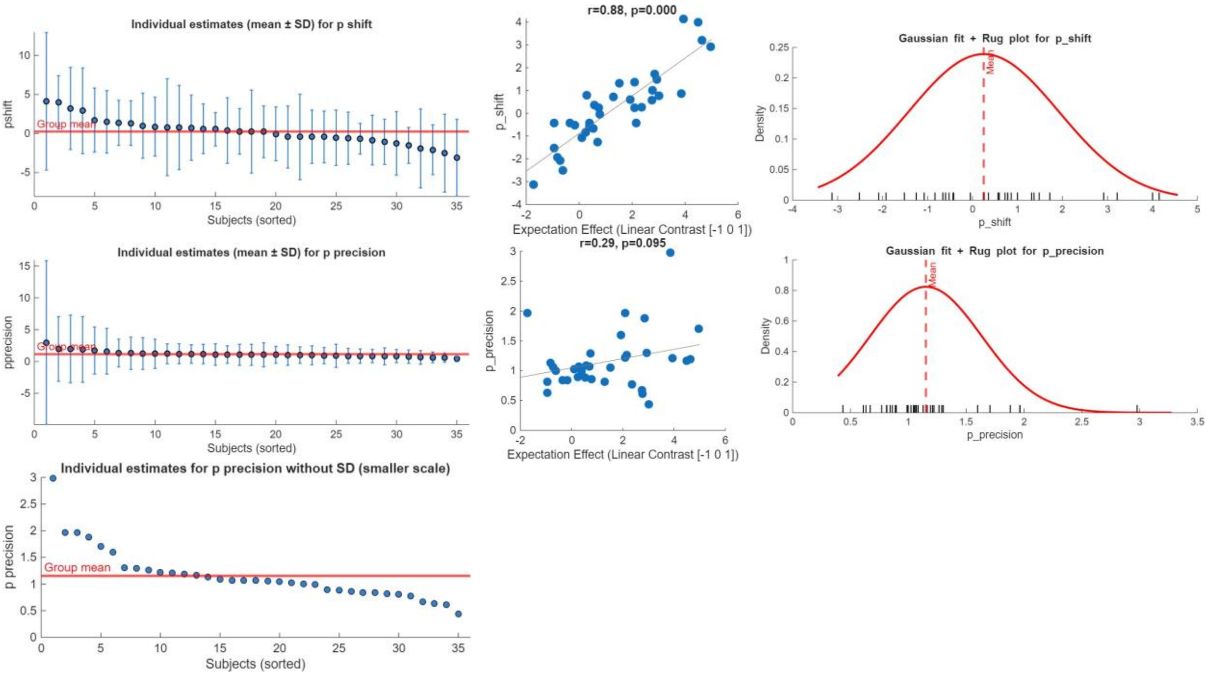

Supplementary Figure 2. Parameter estimates across participants. Parameter estimates across participants. (Top and center row, left) Individual estimates of p_shift (top) and

p_precision (center), sorted in descending order. Each dot represents a participant; error bars indicate the standard deviation (SD) of the posterior parameter estimates, thereby visualizing the posterior density distributions across participants for the estimated parameters. The red line indicates the group mean. To emphasize the interindividual differences in p_precision, we additionally provide a plot with a reduced scale, excluding the variance. (Center column) Correlations between the behavioral expectation effect and the individual parameter estimates for p_shift (top row) and p_precision (Bottom row). (Right column) Gaussian fits of the parameter distributions with rug plots marking individual participants. The red line shows the fitted Gaussian density, and the dashed line indicates the group mean.

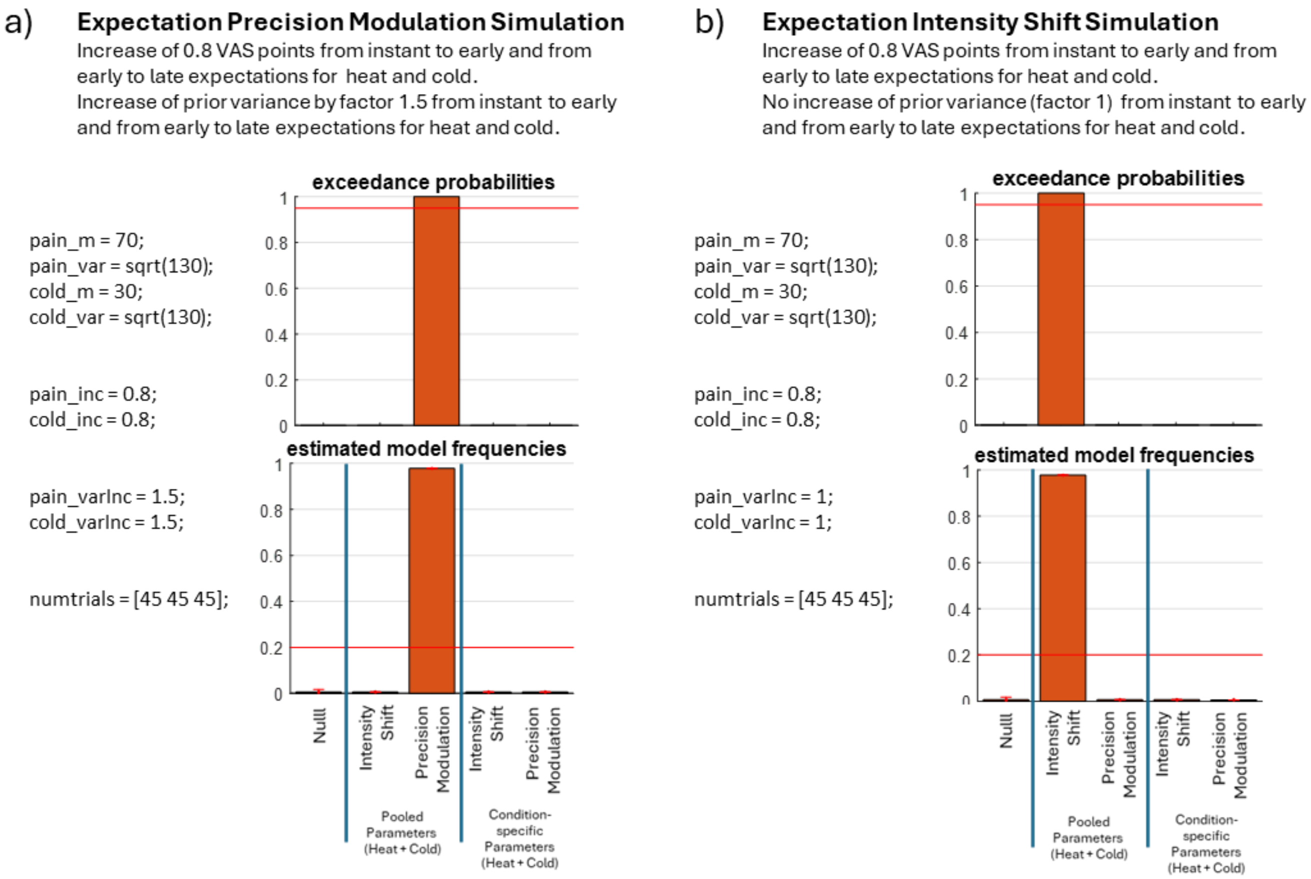

Supplementary Figure 3. Bayesian modelling results with simulated data. In a), we generated a dataset which shows an increase of variance of ratings as well as an increase in of the rated intensity with higher expected delays. Here, the expectation precision modulation model clearly wins in Bayesian model selection. In b), we accordingly generated a dataset without changes in variance with higher expected delays. Here, the expectation intensity shift model clearly wins. Parameters: pain_m/cold_m: mean value of intensity ratings at instant pain stimulation expectation conditions. pain_var/cold_var: variance of intensity ratings at instant pain/cold stimulation expectation conditions. pain_inc/cold_inc: increase/decrease in pain/cold intensity ratings from instant to early and from early to late expectation conditions. pain_varInc/cold_varInc: increase/decrease in pain/cold intensity rating variance from instant to early and from early to late expectation conditions. numtrials: number of trials in instant, early and late conditions.

p_shift mean	p_precision mean	p_offset mean	p_shift SD	p_precision SD	p_offset SD
0,9991	0,6109	0,0148	4,1863	0,7130	31,3974
-0,4194	0,8424	0,0004	3,9834	1,0747	31,6228
4,1502	1,2092	4,5683	8,7993	1,5653	16,0345
-1,2519	1,0669	-0,0005	4,0840	1,6543	31,6228
2,9166	1,7062	2,5292	5,4660	3,7173	4,9874
0,7800	0,4347	-0,0065	5,3806	0,4460	31,5431
0,7839	1,0700	0,0001	6,2063	1,3495	31,6228
0,2507	1,9644	0,0144	5,3217	5,3339	31,4989
-0,6007	0,9892	0,0001	3,3574	1,4820	31,6228
0,5939	1,5995	1,0868	3,1788	3,5730	4,3158
4,0029	1,1633	5,1413	3,4303	1,4817	21,4267
1,7188	1,8817	1,8327	4,1077	5,1260	3,9740
0,7241	0,8119	-0,0011	4,1685	1,0554	31,6226
-0,6612	0,8879	0,0021	1,9862	1,8280	31,6217
-3,1250	1,9659	-0,0061	4,9274	5,0846	31,5957
-1,9240	1,1285	-0,0008	5,0766	1,5295	31,6228
-0,5529	0,8429	0,0117	3,5405	1,0937	31,6010
-0,4329	1,2654	-0,0008	3,3343	2,5019	31,6226
1,3677	1,2220	2,6162	2,9051	2,2879	12,2657
0,3545	1,0854	0,0004	4,1957	1,6643	31,6228
1,4817	1,3018	0,0022	3,9953	2,1605	31,6217
-2,0871	1,0630	-0,0013	3,1978	1,6716	31,6227
-0,4268	0,6330	0,9647	3,4184	0,8300	3,6865
-2,5162	0,9983	0,0000	4,9734	1,2693	31,6228
-0,0489	0,8622	0,0002	3,4191	1,1694	31,6228
1,3138	1,0514	0,0114	2,8583	1,8817	31,6204
0,8594	2,9788	0,8943	3,7716	12,8937	3,1506
-0,4238	1,0450	0,0000	5,4777	1,3313	31,6228
-1,0756	1,0234	0,0000	2,8788	1,6167	31,6228
-1,5162	0,8146	0,0066	2,2850	1,3552	31,5985
-0,8350	0,8959	0,0010	3,1767	1,2907	31,6227
3,2164	1,1912	4,2073	5,2523	1,5530	15,9040
0,2420	1,2889	0,0035	3,3184	2,5015	31,6189
0,2613	0,7698	-0,0047	2,9312	1,0210	31,6104
0,5669	0,6704	0,0032	2,1430	3,3467	31,5971

Supplementary Table 1. Posterior parameter distributions (Gaussian mean ± SD). Reported are the posterior means and standard deviations for p_shift from the expectation intensity shift model and p_precision and p_offset from the expectation precision modulation model.These values provide an explicit summary of the posterior densities (as Gaussian approximations) for each parameter. Note that p_offset shows high SD values (~30) in many cases, hence, we repeated the analysis with fixed p_offset values (with shifts representing 5, 10 and 20) and an “ideal” p_offset value, and in each case, the intensity shift model won.

LME of Behavioral Intensity Ratings with Prediction Errors at Stimulation and Monte Carlo Cluster Analysis for EEG Data for PE at Stimulation

We analyzed trial-wise intensity ratings using a linear mixed-effects (LME) model (as in Results) but replaced the absolute prediction error factor with absolute prediction errors at stimulation, see Figure 1a, bottom row. I.e., we tested the (centered) factor [0.5 0.875 0.875 0.75 0.43 0.86 0.66 0.66 0.33] for [LL-LE (low latency, low expectation) LL-ME LL-HE ML-LE ML-ME ML-HE HL-LE HL-ME HL-HE].

Again, the model revealed a significant main effect of stimulus modality ($t(10072) = 16.88$, $p < .001$, standardized $\beta = 1.69$, SE = 0.10, 95% CI [1.50, 1.89]), i.e. (non-painful) cold stimuli were rated with lower intensities than (painful) heat. We observed a significant main effect of temporal expectations ($t(10072) = 3.21$, $p = .001$, standardized $\beta = 0.02$, SE = 0.006, 95% CI [0.008, 0.033]), indicating that intensity ratings increased with longer expected latency. The actual latency between cue and stimulation did not significantly modulate intensity ratings ($t(10072) = 0.15$, $p = .877$, standardized $\beta = 0.001$, SE = 0.007), i.e., longer latencies were not associated with differences in ratings. We did not observe a significant "prediction error at stimulation factor" effect ($t(10072) = 0.06$, $p = .953$, standardized $\beta < 0.001$, SE = 0.006). Furthermore, no significant interactions of stimulus modality with latency, expectation, or the "prediction error at stimulation factor" were observed (all $p > .76$).

The main effect of temporal prediction errors at stimulation (see Figure 1a, bottom row), was tested with the (centered) factor [0.5 0.875 0.875 0.75 0.43 0.86 0.66 0.66 0.33] for [LL-LE (low latency, low expectation) LL-ME LL-HE ML-LE ML-ME ML-HE HL-LE HL-ME HL-HE] during stimulation. We found an association between stimulus-locked EEG data (0-4s at stimulation onset, 4–181 Hz) with prediction errors at stimulation predominately in beta-to-gamma frequencies (>12 Hz) in the second half of stimulation (see Supplementary Figure 4). Data show a positive cluster of activity, exhibiting a positive association between prediction errors at stimulation and EEG power during stimulation predominately in the beta-to-gamma (>12 Hz) band. This positive cluster ($p < .001$) includes samples ranging from 0 to 4s and frequencies from 4 Hz to 181Hz. The highest parametric F-value from the repeated-measures ANOVA was $F(1,34) = 100.15$ ($p < .001$). This sample was observed at 3.85s and 16 Hz and had a maximum at channel C3.

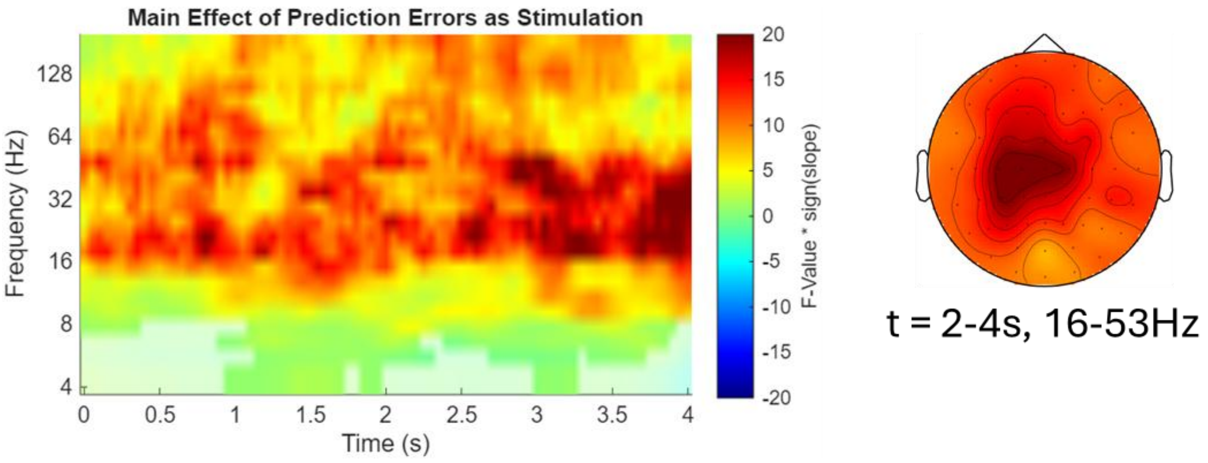

Supplementary Figure 4. Temporal prediction errors at stimulation. The contrast of prediction errors at stimulation (see Figure 1a, bottom row) shows a synchronization of beta-to-gamma frequencies during stimulation presentation. Time-frequency representation with

statistical F-values derived from a repeated measures ANOVA cluster test ($n = 35$) at each sample (time x frequency) averaged over all EEG channels, with $t = 0$ at the onset of the (painful) heat or (non-painful) cold stimulus. Warm colors represent a positive slope of EEG activity (i.e. a higher power was detected with increased prediction errors), whereas cold colors represent a negative slope (i.e. a lower power with increased prediction errors). EEG power was averaged within participants before group-level statistics. Significant clusters are highlighted. Topographies represent F-values. P-value of significant cluster: $p < .001$.

Monte Carlo Cluster Analysis for EEG Data for PE only with a trial count ≥ 10

To address the concern that some participants contributed fewer than 10 trials particularly in high PE conditions, we repeated the prediction error (PE) time–frequency analysis including only those participants who had at least 10 trials in the high-PE condition, resulting in a remaining N = 30.

For the main effect of (absolute) temporal prediction errors (Supplementary Figure 5), we found an association between stimulus-locked EEG data for subjects with a trial count ≥ 10 (0-4s at stimulation onset, 4–181 Hz) with absolute temporal prediction errors in beta-to-gamma frequencies (>12 Hz) predominately in the second half of stimulation (>30 Hz; Figure 8). This positive cluster ($p < .001$) includes samples ranging from 0 to 4s and frequencies from >5.6 Hz. The highest parametric F-value from the repeated-measures ANOVA was $F(1,34) = 74.13$ ($p < .001$). This sample was observed at 2.8s and 53.79 Hz and had a maximum at channel FC3. We found no significant interaction between absolute prediction errors and stimulus modality (all $p > .05$).

This reduced dataset reproduced the same result as the full sample: beta–gamma (>12Hz) activity remained positively associated with PE. Thus, the main finding is not driven by participants with lower trial counts.

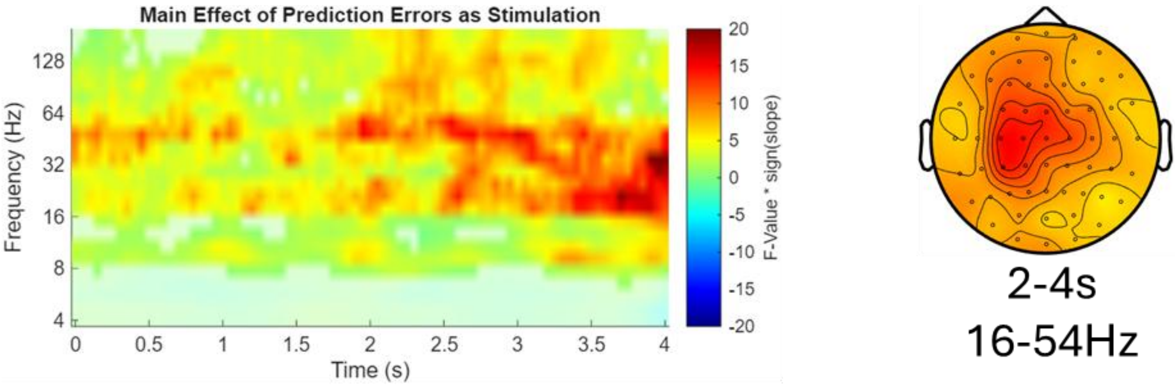

Supplementary Figure 5. Temporal prediction errors with trial count > 10. The contrast of absolute prediction errors (i.e. the absolute difference between expected and actual cue-stimulus delay) shows a synchronization of beta-to-gamma frequencies during stimulation presentation. Time-frequency representation with statistical F-values derived from a repeated measures ANOVA cluster test (n = 35) at each sample (time x frequency) averaged over all EEG channels, with t = 0 at the onset of the (painful) heat or (non-painful) cold stimulus. Warm colors represent a positive slope of EEG activity (i.e. a higher power was detected with increased absolute prediction errors), whereas cold colors represent a negative slope (i.e. a lower power with increased absolute prediction errors). EEG power was averaged within participants before group-level statistics. Topographies represent F-values at each electrode within respective frequency bands in significant time frames. P-value of significant cluster: $p < .001$.

Prediction Errors at Instant, Early and Late Stimulation

To explore how prediction errors evolve over time, we conducted F-tests at each sample (channel x time frequency) from 0-4s for instant stimulation with a factor which represents if prediction errors are present, i.e. [0 1 1], where 0 represents instant stimulation expectations, and 1 represents early stimulation and late stimulation expectations (representing a PE). Note that this is not orthogonal to an expectation effect, which would be coded [0 1 2]. For early stimulation, we conducted F-tests with factor levels [1 0 1], here, 1 represents a prediction error elicited by instant and late expectations, whereas early expectations do not lead to a prediction error. For late stimulation, we conducted F-tests with factor levels [1 1 0], here, instant and early stimulation are associated with a prediction error, while late stimulation has no prediction errors. Note, again, that this is not orthogonal to an expectation effect.

Results show that for all latencies, prediction errors are represented during stimulation, i.e. during instant stimulation (0-4s), early stimulation (2-6s) and late stimulation (4-8s) (see Supplementary Figure 6).

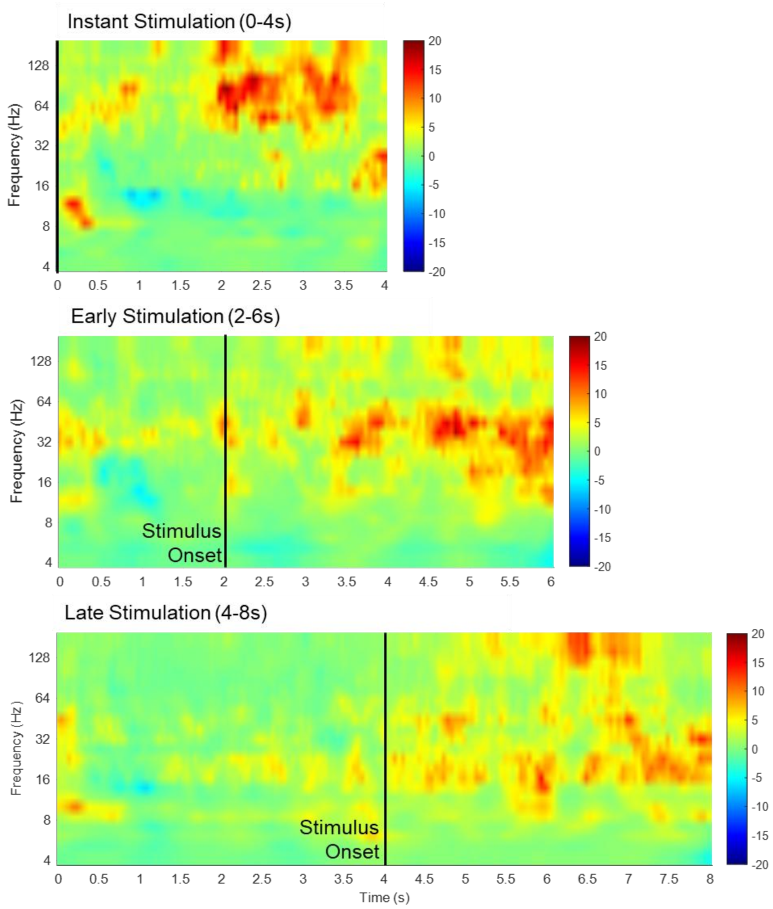

Supplementary Figure 6. Time-frequency patterns of absolute prediction errors over time (Note: in instant and late stimulation the PE factor is not orthogonal to an expectation factor).

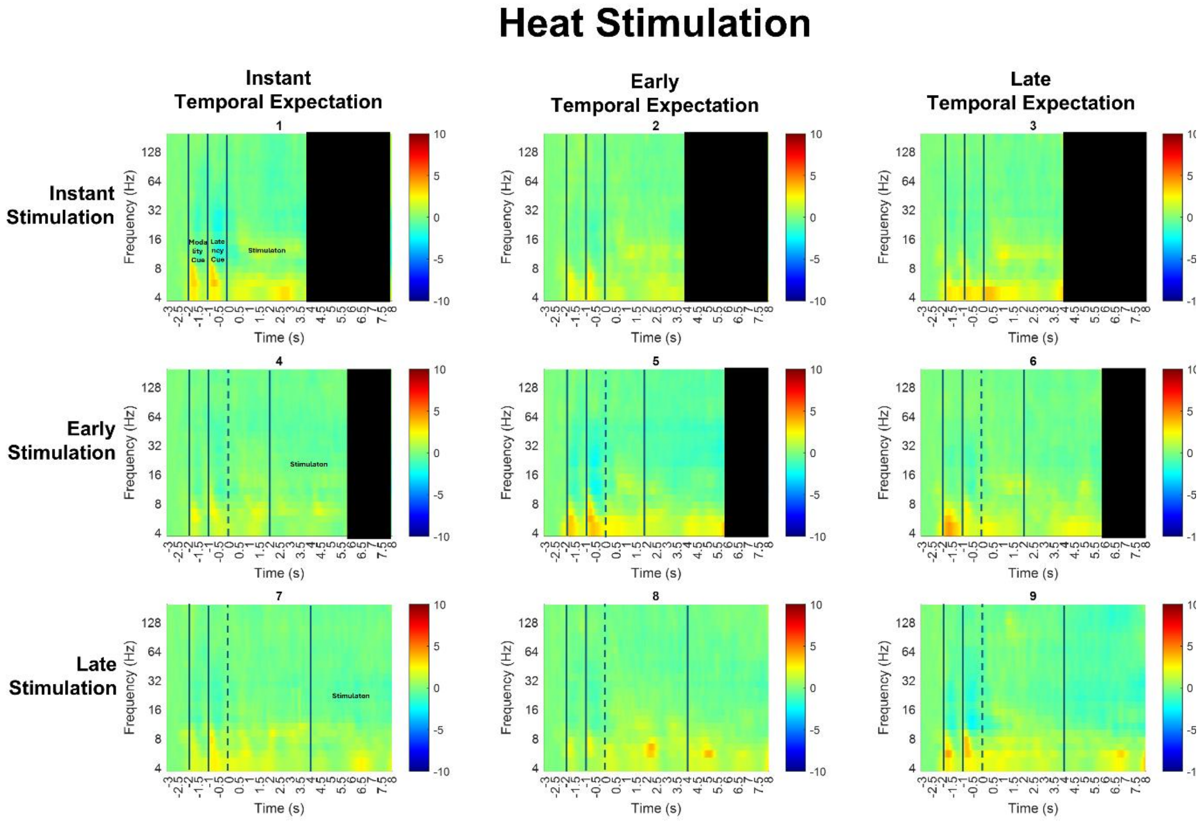

Supplementary Figure 7. Time-frequency patterns of z-standardized (baseline -3 to -2.5s) EEG power over time for each condition with pain stimulation.

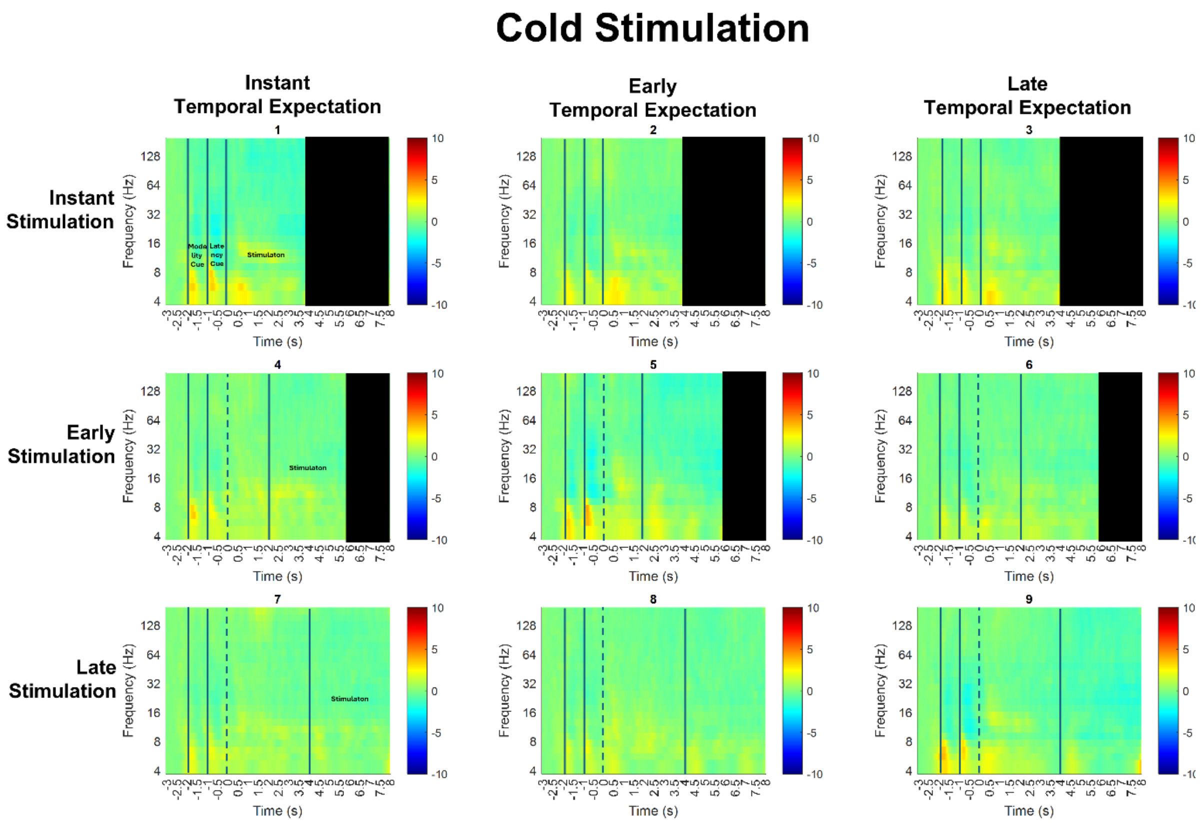

Supplementary Figure 8. Time-frequency patterns of z-standardized (baseline -3 to -2.5s) EEG power over time for each condition with cold stimulation.

	LE-LL Heat		ME-LL Heat		HE-LL Heat		LE-ML Heat		ME-ML Heat		HE-ML Heat		LE-HL Heat		ME-HL Heat		HE-HL Heat		LE-Catch Heat		ME-Catch Heat		HE-Catch Heat	
**Subject**	LF	HF	LF	HF	LF	HF	LF	HF	LF	HF	LF	HF	LF	HF	LF	HF	LF	HF	LF	HF	LF	HF	LF	HF
1	31	29	6	6	6	6	6	6	29	27	4	4	7	7	7	6	26	24	12	14	15	12	12	12
2	28	24	8	7	8	7	8	7	31	19	7	7	8	7	8	6	28	24	16	10	16	15	15	15
3	27	29	7	7	8	8	8	7	27	29	6	6	6	6	6	7	31	28	15	13	16	16	15	16
4	29	13	8	3	8	4	7	5	28	18	7	3	8	4	8	4	32	23	15	8	16	13	16	13
5	29	25	8	6	8	7	7	8	31	29	7	7	8	5	8	4	29	27	16	15	16	15	15	15
6	24	25	8	8	7	7	7	5	30	26	7	7	8	7	7	7	29	23	14	8	15	14	14	14
7	32	19	8	5	8	6	8	5	31	18	8	4	8	6	8	5	31	20	14	9	15	10	16	10
8	32	32	8	7	8	8	8	7	29	31	7	6	7	7	8	8	29	28	14	16	16	16	16	16
9	23	26	6	7	6	6	6	8	24	29	6	7	6	6	6	8	24	28	11	13	11	14	11	14
10	32	28	8	8	8	7	8	8	31	29	8	7	8	7	8	7	29	28	14	11	15	15	15	15
11	29	29	8	7	8	8	7	8	28	31	8	8	8	8	8	8	31	31	16	16	15	15	15	15
12	25	20	6	6	6	4	7	7	24	19	7	6	6	6	7	6	25	21	14	11	16	15	13	15
13	31	32	7	7	8	8	7	8	29	30	7	8	6	7	7	7	29	31	16	16	14	15	14	15
14	30	28	8	8	8	7	7	6	29	27	8	8	7	5	7	7	28	24	15	15	14	14	13	14
15	27	24	8	6	7	6	6	7	29	23	8	7	8	6	7	8	31	29	16	13	14	10	13	10
16	29	26	7	6	7	7	6	6	31	28	7	6	6	5	8	7	31	30	15	16	16	15	14	15
17	32	32	8	8	8	8	8	7	31	31	6	6	8	7	6	7	32	32	15	15	16	16	15	16
18	29	31	6	7	8	8	7	7	30	31	8	8	6	7	8	8	27	32	16	16	16	16	16	16
19	30	26	8	7	8	8	6	6	30	29	8	6	8	8	6	4	28	24	16	15	14	12	15	12
20	26	20	4	4	7	5	7	5	30	20	8	6	7	7	7	8	30	18	15	13	15	11	15	11
21	31	26	7	7	7	4	8	7	32	24	8	8	8	7	7	7	31	29	14	13	15	14	16	14
22	31	31	8	7	7	7	8	6	32	30	8	8	8	7	7	7	31	31	15	14	16	14	15	14
23	30	20	7	7	7	6	8	7	32	24	8	8	6	6	8	5	30	27	15	12	16	13	16	13
24	31	30	8	8	7	7	8	8	30	29	8	8	8	7	8	7	32	30	14	15	15	14	16	14
25	28	15	8	4	8	4	8	5	32	18	6	2	7	4	6	3	28	18	16	9	16	11	15	11
26	32	20	8	7	8	7	8	7	32	28	8	8	8	8	8	7	32	26	16	15	15	12	16	12
27	30	27	7	7	7	7	8	8	31	29	8	7	8	8	8	6	32	27	15	13	15	14	16	14
28	31	17	8	5	8	5	8	7	30	23	8	5	7	7	8	6	32	20	16	11	16	13	15	13
29	32	30	8	8	8	8	8	8	32	28	8	8	8	6	8	8	31	29	16	16	14	14	16	14
30	27	22	7	6	8	8	7	6	29	27	5	5	6	4	7	5	28	28	13	14	13	10	15	10
31	29	25	8	8	8	7	7	6	30	28	8	8	8	8	8	5	32	28	16	16	16	14	15	14
32	29	25	8	6	7	6	8	7	28	27	8	7	6	6	8	8	29	29	15	14	14	11	16	11
33	27	28	7	7	8	8	8	8	30	26	8	8	8	8	7	8	25	25	14	14	14	14	15	14
34	26	25	8	7	8	8	7	7	31	28	6	6	8	7	6	6	31	26	15	15	13	14	16	14
35	24	17	6	4	7	5	8	7	29	27	7	8	7	5	7	7	26	23	15	12	12	11	14	11
**Avg**	**29**	**25**	**7,4**	**6,5**	**7,5**	**6,6**	**7,4**	**6,8**	**30**	**26**	**7,3**	**6,6**	**7,3**	**6,5**	**7,3**	**6,5**	**29**	**26**	**15**	**13**	**15**	**13**	**15**	**13**
Min	23	13	4	3	6	4	6	5	24	18	4	2	6	4	6	3	24	18	11	8	11	10	11	10
Max	32	32	8	8	8	8	8	8	32	31	8	8	8	8	8	8	32	32	16	16	16	16	16	16

Supplementary Table 2. Number of trials after data cleaning in heat modality conditions for each participant. LE = Low Expectation, ME = Medium Expectation, HE = High Expectation, LL = Low Latency, ML = Medium Latency, HL = High Latency, LF = Low Frequency and HF = High Frequency.

	LE-LL Cold		ME-LL Cold		HE-LL Cold		LE-ML Cold		ME-ML Cold		HE-ML Cold		LE-HL Cold		ME-HL Cold		HE-HL Cold		LE-Catch Cold		ME-Catch Cold		HE-Catch Cold	
**Subject**	LF	HF	LF	HF	LF	HF	LF	HF	LF	HF	LF	HF	LF	HF	LF	HF	LF	HF	LF	HF	LF	HF	LF	HF
1	28	26	7	8	6	7	7	6	25	23	7	7	7	7	8	8	27	25	15	15	13	14	15	14
2	28	23	7	4	8	3	8	6	28	22	7	5	7	5	8	5	31	25	14	12	16	14	16	13
3	30	28	8	7	6	8	8	8	30	31	8	7	7	7	8	8	31	32	16	15	16	16	14	16
4	30	20	7	6	8	6	8	6	32	14	8	6	8	6	8	6	28	23	16	11	14	7	16	12
5	32	29	8	7	8	6	8	7	32	26	8	7	7	7	7	7	30	25	15	11	16	15	15	14
6	30	28	8	7	8	8	7	7	31	26	8	6	8	6	7	6	32	29	15	15	15	14	14	14
7	32	17	8	6	8	3	8	5	28	20	7	5	8	5	8	6	31	15	16	11	16	8	14	11
8	29	29	6	6	8	7	8	8	30	30	8	8	8	8	8	8	32	30	16	15	14	13	16	15
9	23	27	5	7	4	6	6	7	24	25	6	7	6	7	6	7	24	27	12	16	11	13	12	14
10	30	27	8	7	7	6	8	7	30	30	8	7	8	7	8	8	30	30	16	15	16	15	15	15
11	29	30	8	8	8	8	8	8	30	30	7	8	8	8	7	8	31	32	16	16	13	16	15	14
12	30	27	7	7	6	5	8	7	31	29	8	7	7	7	8	7	29	28	15	13	16	13	16	13
13	30	29	8	8	7	7	8	7	27	32	7	8	7	8	8	8	28	31	15	16	15	15	16	16
14	28	27	7	7	7	7	8	8	30	29	8	7	6	7	8	8	30	29	15	15	14	15	14	14
15	28	24	8	7	7	6	7	6	32	26	8	5	8	5	8	7	31	26	16	14	15	14	16	16
16	31	29	7	6	6	6	8	8	32	28	7	7	8	7	6	4	28	25	14	13	15	12	14	14
17	31	31	8	8	8	8	8	8	32	32	6	6	8	6	8	8	31	32	14	14	16	15	16	16
18	32	32	8	8	8	8	7	8	28	30	8	8	8	8	8	8	32	31	15	16	16	16	16	16
19	31	29	7	6	8	6	7	7	30	27	8	7	7	7	7	7	31	25	16	16	15	12	16	16
20	26	19	8	6	7	4	7	6	25	19	6	7	8	7	6	5	28	22	14	9	14	8	14	12
21	32	24	8	8	8	7	8	6	32	27	8	7	8	7	7	6	29	24	16	14	16	15	16	12
22	32	23	8	8	8	7	7	7	31	32	8	8	8	8	8	8	31	30	16	13	16	14	15	14
23	30	24	8	6	8	7	6	6	31	27	8	6	8	6	8	7	29	27	16	13	14	10	15	13
24	32	32	8	8	8	6	8	8	29	29	8	8	7	8	8	8	32	32	13	14	16	15	15	15
25	31	20	8	5	8	5	8	5	30	24	8	4	7	4	8	7	32	23	16	10	15	9	16	12
26	31	28	8	8	8	8	8	8	32	32	8	6	8	6	7	7	31	28	16	14	16	14	15	13
27	32	28	8	6	8	6	8	6	32	26	8	7	8	7	8	7	31	29	16	13	15	16	16	13
28	30	20	8	6	8	5	8	7	28	16	6	4	8	4	7	2	31	28	15	10	16	10	16	9
29	29	28	7	7	8	8	8	8	32	31	8	7	8	7	7	7	32	29	16	15	14	14	14	16
30	29	28	8	7	8	7	7	7	27	25	6	5	8	5	7	7	31	31	15	14	13	14	12	15
31	32	31	8	5	8	7	7	7	31	25	8	6	8	6	8	8	29	28	14	13	13	12	15	15
32	30	26	8	7	8	7	7	6	30	26	8	6	7	6	7	8	30	29	16	14	14	14	15	16
33	27	27	6	7	7	7	7	6	30	30	7	7	7	7	8	8	31	31	15	15	16	16	15	15
34	27	26	8	8	8	7	6	6	28	28	7	7	7	7	5	5	29	28	16	13	14	14	15	14
35	28	27	7	8	7	7	5	5	27	27	3	6	7	6	6	7	26	29	14	12	14	10	13	12
**Avg**	**30**	**26**	**7,5**	**6,9**	**7,5**	**6,5**	**7,4**	**6,8**	**30**	**27**	**7,3**	**6,5**	**7,5**	**6,5**	**7,4**	**6,9**	**30**	**28**	**15**	**14**	**15**	**13**	**15**	**14**
Min	23	17	5	4	4	3	5	5	24	14	3	4	6	4	5	2	24	15	12	9	11	7	12	9
Max	32	32	8	8	8	8	8	8	32	32	8	8	8	8	8	8	32	32	16	16	16	16	16	16

Supplementary Table 3. Number of trials after data cleaning in cold modality conditions for each participant. LE = Low Expectation, ME = Medium Expectation, HE = High Expectation, LL = Low Latency, ML = Medium Latency, HL = High Latency, LF = Low Frequency and HF = High Frequency.

	Instant Stim		Early Stim		Late Stim		Instant Exp		Early Exp		Late Exp		No PE		Medium PE		High PE	
**Subject**	LF	HF	LF	HF	LF	HF	LF	HF	LF	HF	LF	HF	LF	HF	LF	HF	LF	HF
1	43	41	39	37	40	37	44	42	42	39	36	34	86	80	23	22	13	13
2	44	38	46	33	44	37	44	38	47	32	43	38	87	67	31	27	16	14
3	42	44	41	42	43	41	41	42	40	43	45	42	85	86	27	27	14	14
4	45	20	42	26	48	31	44	22	44	25	47	30	89	54	30	15	16	8
5	45	38	45	44	45	36	44	38	47	39	44	41	89	81	30	25	16	12
6	39	40	44	38	44	37	39	37	45	41	43	37	83	74	29	27	15	14
7	48	30	47	27	47	31	48	30	47	28	47	30	94	57	32	19	16	12
8	48	47	44	44	44	43	47	46	45	46	44	42	90	91	31	28	15	15
9	35	39	36	44	36	42	35	40	36	44	36	41	71	83	24	30	12	12
10	48	43	47	44	45	42	48	43	47	44	45	42	92	85	32	30	16	14
11	45	44	43	47	47	47	44	45	44	46	47	47	88	91	31	31	16	16
12	37	30	38	32	38	33	38	33	37	31	38	31	74	60	27	25	12	10
13	46	47	43	46	42	45	44	47	43	44	44	47	89	93	28	30	14	15
14	46	43	44	41	42	36	44	39	44	42	44	39	87	79	30	29	15	12
15	42	36	43	37	46	43	41	37	44	37	46	42	87	76	29	28	15	12
16	43	39	44	40	45	42	41	37	46	41	45	43	91	84	28	25	13	12
17	48	48	45	44	46	46	48	46	45	46	46	46	95	95	28	28	16	15
18	43	46	45	46	41	47	42	45	44	46	43	48	86	94	29	30	14	15
19	46	41	44	41	42	36	44	40	44	40	44	38	88	79	28	23	16	16
20	37	29	45	31	44	33	40	32	41	32	45	29	86	58	26	23	14	12
21	45	37	48	39	46	43	47	40	46	38	46	41	94	79	30	29	15	11
22	46	45	48	44	46	45	47	44	47	44	46	46	94	92	31	28	15	14
23	44	33	48	39	44	38	44	33	47	36	45	41	92	71	31	27	13	12
24	46	45	46	45	48	44	47	45	46	44	47	45	93	89	32	31	15	14
25	44	23	46	25	41	25	43	24	46	25	42	24	88	51	28	14	15	8
26	48	34	48	43	48	41	48	35	48	42	48	41	96	74	32	29	16	15
27	44	41	47	44	48	41	46	43	46	42	47	41	93	83	31	28	15	15
28	47	27	46	35	47	33	46	31	46	34	48	30	93	60	32	23	15	12
29	48	46	48	44	47	43	48	44	48	44	47	45	95	87	32	32	16	14
30	42	36	41	38	41	37	40	32	43	38	41	41	84	77	26	22	14	12
31	45	40	45	42	48	41	44	39	46	41	48	43	91	81	31	27	16	15
32	44	37	44	41	43	43	43	38	44	41	44	42	86	81	32	28	13	12
33	42	43	46	42	40	41	43	44	44	41	41	41	82	79	30	31	16	16
34	42	40	44	41	45	39	41	39	45	41	45	40	88	79	27	26	16	15
35	37	26	44	42	40	35	39	29	42	38	40	36	79	67	28	26	14	10
Avg	**44**	**38**	**44**	**40**	**44**	**39**	**44**	**38**	**44**	**39**	**44**	**40**	**88**	**78**	**29**	**26**	**15**	**13**
Min	35	20	36	25	36	25	35	22	36	25	36	24	71	51	23	14	12	8
Max	48	48	48	47	48	47	48	47	48	46	48	48	96	95	32	32	16	16

Supplementary Table 4. Number of trials for each experimental condition after data cleaning in heat modality conditions for each participant. LF = Low Frequency and HF = High Frequency.

	Instant Stim		Early Stim		Late Stim		Instant Exp		Early Exp		Late Exp		No PE		Medium PE		High PE	
**Subject**	LF	HF	LF	HF	LF	HF	LF	HF	LF	HF	LF	HF	LF	HF	LF	HF	LF	HF
1	41	41	39	36	42	40	42	39	40	39	40	39	80	74	29	29	13	14
2	43	30	43	33	46	35	43	34	43	31	46	33	87	70	30	20	15	8
3	44	43	46	46	46	47	45	43	46	46	45	47	91	91	32	30	13	15
4	45	32	48	26	44	35	46	32	47	26	44	35	90	57	31	24	16	12
5	48	42	48	40	44	39	47	43	47	40	46	38	94	80	31	28	15	13
6	46	43	46	39	47	41	45	41	46	39	48	43	93	83	30	26	16	14
7	48	26	43	30	47	26	48	27	44	32	46	23	91	52	31	22	16	8
8	43	42	46	46	48	46	45	45	44	44	48	45	91	89	30	30	16	15
9	32	40	36	39	36	41	35	41	35	39	34	40	71	79	23	28	10	13
10	45	40	46	44	46	45	46	41	46	45	45	43	90	87	32	29	15	13
11	45	46	45	46	46	48	45	46	45	46	46	48	90	92	30	32	16	16
12	43	39	47	43	44	42	45	41	46	43	43	40	90	84	31	28	13	12
13	45	44	42	47	43	47	45	44	43	48	42	46	85	92	31	31	14	15
14	42	41	46	44	44	44	42	42	45	44	45	43	88	85	31	30	13	14
15	43	37	47	37	47	38	43	35	48	40	46	37	91	76	31	25	15	11
16	44	41	47	43	42	36	47	44	45	38	41	38	91	82	28	25	14	13
17	47	47	46	46	47	46	47	45	48	48	45	46	94	95	30	30	16	14
18	48	48	43	46	48	47	47	48	44	46	48	47	92	93	31	32	16	16
19	46	41	45	41	45	39	45	43	44	40	47	38	92	81	29	27	15	13
20	41	29	38	32	42	34	41	32	39	30	41	33	79	60	27	24	15	11
21	48	39	48	40	44	37	48	37	47	41	45	38	93	75	31	27	16	14
22	48	38	46	47	47	46	47	38	47	48	47	45	94	85	31	31	16	15
23	46	37	45	39	45	40	44	36	47	40	45	40	90	78	30	25	16	13
24	48	46	45	45	47	48	47	48	45	45	48	46	93	93	32	32	15	14
25	47	30	46	33	47	34	46	29	46	36	48	32	93	67	32	21	15	9
26	47	44	48	46	46	41	47	42	47	47	47	42	94	88	31	29	16	14
27	48	40	48	39	47	43	48	41	48	39	47	42	95	83	32	26	16	13
28	46	31	42	27	46	34	46	31	43	24	45	37	89	64	29	19	16	9
29	44	43	48	46	47	43	45	43	46	45	48	44	93	88	30	29	16	15
30	45	42	40	37	46	43	44	40	42	39	45	43	87	84	28	26	16	12
31	48	43	46	38	45	42	47	44	47	38	45	41	92	84	31	26	16	13
32	46	40	45	38	44	43	44	38	45	41	46	42	90	81	30	27	15	13
33	40	41	44	43	46	46	41	40	44	45	45	45	88	88	28	28	14	14
34	43	41	41	41	41	40	40	39	41	41	44	42	84	82	26	26	15	14
35	42	42	35	38	39	42	40	38	40	42	36	42	81	83	21	26	14	13
Avg	**45**	**40**	**44**	**40**	**45**	**41**	**45**	**40**	**45**	**40**	**45**	**41**	**89**	**81**	**30**	**27**	**15**	**13**
Min	32	26	35	26	36	26	35	27	35	24	34	23	71	52	21	19	10	8
Max	48	48	48	47	48	48	48	48	48	48	48	48	95	95	32	32	16	16

Supplementary Table 5. Number of trials for each experimental condition after data cleaning in cold modality conditions for each participant. LF = Low Frequency and HF = High Frequency.
