# Supplementary material for: Temporal predictions shape somatosensory perception
Source: Nat Commun. 2026 Apr 14;17:3476. doi: 10.1038/s41467-026-71600-y (PMC13079782; doi:10.1038/s41467-026-71600-y)
Supplement: Supplementary file 2 — Reporting Summary [file 41467_2026_71600_MOESM2_ESM.pdf]

nature portfolio

Corresponding author(s): Andreas Strube, Christian Büchel

Last updated by author(s): 03/12/2026

Reporting Summary

Nature Portfolio wishes to improve the reproducibility of the work that we publish. This form provides structure for consistency and transparency in reporting. For further information on Nature Portfolio policies, see our Editorial Policies and the Editorial Policy Checklist.

Statistics

For all statistical analyses, confirm that the following items are present in the figure legend, table legend, main text, or Methods section.

n/a	Confirmed	
☐	☒	The exact sample size (n) for each experimental group/condition, given as a discrete number and unit of measurement
☐	☒	A statement on whether measurements were taken from distinct samples or whether the same sample was measured repeatedly
☐	☒	The statistical test(s) used AND whether they are one- or two-sided<br>*Only common tests should be described solely by name; describe more complex techniques in the Methods section.*
☐	☒	A description of all covariates tested
☐	☒	A description of any assumptions or corrections, such as tests of normality and adjustment for multiple comparisons
☐	☒	A full description of the statistical parameters including central tendency (e.g. means) or other basic estimates (e.g. regression coefficient) AND variation (e.g. standard deviation) or associated estimates of uncertainty (e.g. confidence intervals)
☐	☒	For null hypothesis testing, the test statistic (e.g. F, t, r) with confidence intervals, effect sizes, degrees of freedom and P value noted<br>*Give P values as exact values whenever suitable.*
☐	☒	For Bayesian analysis, information on the choice of priors and Markov chain Monte Carlo settings
☒	☐	For hierarchical and complex designs, identification of the appropriate level for tests and full reporting of outcomes
☐	☒	Estimates of effect sizes (e.g. Cohen's d, Pearson's r), indicating how they were calculated

Our web collection on statistics for biologists contains articles on many of the points above.

Software and code

Policy information about availability of computer code

Data collection	BrainVision Recorder<br>Matlab 2022a/2022b<br>Psychophysics Toolbox
Data analysis	Matlab 2025a<br>Fieldtrip 20240110<br>VBA Toolbox (v04/05/2019)

For manuscripts utilizing custom algorithms or software that are central to the research but not yet described in published literature, software must be made available to editors and reviewers. We strongly encourage code deposition in a community repository (e.g. GitHub). See the Nature Portfolio guidelines for submitting code & software for further information.

Data

Policy information about availability of data

All manuscripts must include a data availability statement. This statement should provide the following information, where applicable:

- Accession codes, unique identifiers, or web links for publicly available datasets
- A description of any restrictions on data availability
- For clinical datasets or third party data, please ensure that the statement adheres to our policy

Data for this study are publicly available on https://osf.io/tajch/. Project DOI: 10.17605/OSF.IO/TAJCH

Research involving human participants, their data, or biological material

Policy information about studies with human participants or human data. See also policy information about sex, gender (identity/presentation), and sexual orientation and race, ethnicity and racism.

Reporting on sex and gender	Participants were asked to indicate their sex (German: Geschlecht) and the sample included 17 male and 19 female participants. As our study focused on basic perceptual and neural mechanisms, we did not explicitly test for sex or gender differences. While we do not expect substantial differences in the context of the investigated processes, identifying potential sex- or gender-related effects would likely require larger, specifically powered samples and targeted hypotheses.
Reporting on race, ethnicity, or other socially relevant groupings	Information on race, ethnicity or other social grouping was not collected in this study. As the study focused on basic perceptual and neural mechanisms, no group-specific hypotheses were formulated. We acknowledge the importance of diversity in research samples and encourage future studies to explore potential sociocultural influences where relevant.
Population characteristics	See above.
Recruitment	Participants were recruited via a German job portal.
Ethics oversight	Ethics comittee of the Hamburg Medical Association.

Note that full information on the approval of the study protocol must also be provided in the manuscript.

Field-specific reporting

Please select the one below that is the best fit for your research. If you are not sure, read the appropriate sections before making your selection.

☐ Life sciences ☒ Behavioural & social sciences ☐ Ecological, evolutionary & environmental sciences

For a reference copy of the document with all sections, see nature.com/documents/nr-reporting-summary-flat.pdf

Behavioural & social sciences study design

All studies must disclose on these points even when the disclosure is negative.

Study description	Quantitative Date (EEG + Behavior)
Research sample	The sample size was determined through a priori power analysis using G*Power (version 3.1.9.4). We informed the power analysis by behavioral effects observed in Strube et al. (2023), where expectations were manipulated under similar experimental conditions in the same laboratory. That study reported large effect sizes (Cohen's f > 0.5) for both intensity expectation effects and modulations of pain ratings by agency. Assuming a low correlation among repeated measures (r = 0.2), an alpha level of 0.05, and a desired power of 0.80, the analysis indicated that a sample size of 12 subjects would be sufficient to detect similar effects. However, considering potential differences in expectation modulation - specifically, temporal versus intensity expectations - we increased the planned sample size to 35 subjects. Accounting for an anticipated dropout of four subjects, a final sample size of 31 provides adequate power to detect medium effect sizes (f = 0.3) in our within-subject design.
Sampling strategy	Participants were recruited via convenience sampling using advertisements on a local job platform, targeting the local community and university population. Potential self-selection effects are inherent to this recruitment strategy.
Data collection	Computer / BrainVision EEG System / Informed Consent / EEG preparation / 3-4 hour duration
Timing	05/08/2022-10/18/2023
Data exclusions	No data was excluded from analysis.
Non-participation	No participant dropped out.

Randomization	Partcipants were not allocated to experimental groups (full within subject design).

Reporting for specific materials, systems and methods

We require information from authors about some types of materials, experimental systems and methods used in many studies. Here, indicate whether each material, system or method listed is relevant to your study. If you are not sure if a list item applies to your research, read the appropriate section before selecting a response.

Materials & experimental systems

n/a	Involved in the study
☒	☐ Antibodies
☒	☐ Eukaryotic cell lines
☒	☐ Palaeontology and archaeology
☒	☐ Animals and other organisms
☒	☐ Clinical data
☒	☐ Dual use research of concern
☒	☐ Plants

Methods

n/a	Involved in the study
☒	☐ ChIP-seq
☒	☐ Flow cytometry
☒	☐ MRI-based neuroimaging

Plants

Seed stocks	*Report on the source of all seed stocks or other plant material used. If applicable, state the seed stock centre and catalogue number. If plant specimens were collected from the field, describe the collection location, date and sampling procedures.*
Novel plant genotypes	*Describe the methods by which all novel plant genotypes were produced. This includes those generated by transgenic approaches, gene editing, chemical/radiation-based mutagenesis and hybridization. For transgenic lines, describe the transformation method, the number of independent lines analyzed and the generation upon which experiments were performed. For gene-edited lines, describe the editor used, the endogenous sequence targeted for editing, the targeting guide RNA sequence (if applicable) and how the editor was applied.*
Authentication	*Describe any authentication procedures for each seed stock used or novel genotype generated. Describe any experiments used to assess the effect of a mutation and, where applicable, how potential secondary effects (e.g. second site T-DNA insertions, mosiacism, off-target gene editing) were examined.*
